# Accurate and Robust Alignment of Differently Stained Histologic Images Based on Greedy Diffeomorphic Registration

**DOI:** 10.3390/app11041892

**Published:** 2021-02-21

**Authors:** Ludovic Venet, Sarthak Pati, Michael D. Feldman, MacLean P. Nasrallah, Paul Yushkevich, Spyridon Bakas

**Affiliations:** 1 Center for Biomedical Image Computing & Analytics, University of Pennsylvania, Philadelphia, PA 19104, USA;; 2 Department of Radiology, Perelman School of Medicine, University of Pennsylvania, Philadelphia, PA 19104, USA; 3 Department of Pathology & Laboratory Medicine, Perelman School of Medicine, University of Pennsylvania, Philadelphia, PA 19104, USA;

**Keywords:** registration, deformable, diffeomorphic, digital pathology, histology, histopathology, ANHIR challenge

## Abstract

Histopathologic assessment routinely provides rich microscopic information about tissue structure and disease process. However, the sections used are very thin, and essentially capture only 2D representations of a certain tissue sample. Accurate and robust alignment of sequentially cut 2D slices should contribute to more comprehensive assessment accounting for surrounding 3D information. Towards this end, we here propose a two-step diffeomorphic registration approach that aligns differently stained histology slides to each other, starting with an initial affine step followed by estimating a deformation field. It was quantitatively evaluated on ample (*n* = 481) and diverse data from the automatic non-rigid histological image registration challenge, where it was awarded the second rank. The obtained results demonstrate the ability of the proposed approach to robustly (average robustness = 0.9898) and accurately (average relative target registration error = 0.2%) align differently stained histology slices of various anatomical sites while maintaining reasonable computational efficiency (<1 min per registration). The method was developed by adapting a general-purpose registration algorithm designed for 3D radiographic scans and achieved consistently accurate results for aligning high-resolution 2D histologic images. Accurate alignment of histologic images can contribute to a better understanding of the spatial arrangement and growth patterns of cells, vessels, matrix, nerves, and immune cell interactions.

## 1. Introduction

Histologic and, more recently, immunohistochemical evaluation of resected tissue by anatomic pathologists, are the essential basis of surgical pathology diagnostics. Variously stained histology slices are routinely used by pathologists to assess tissue samples from various anatomical sites and determine tissue structure, the presence or extent of a disease, as well as the host reaction that describes the disease process. However, as the field continues to move forward, new technologies in imaging, protein, and nucleic acid analysis will enhance these traditional assessment techniques to allow more precise and actionable diagnoses [1]. This phenomenon has been dramatically exemplified by the integration of molecular features into diagnostic criteria. Similarly, rich data reflecting the biology underlying various pathologic processes are obtained by leveraging advances in imaging and machine learning in order to analyze histopathology slides to elucidate imaging features in a quantitative and reproducible manner. These structural correlates of biological processes, particularly in the context of molecular insight when available, may lead to improved ability to tailor therapy based on biological markers.

Non-rigid registration of consecutive 2D histologic slices with different stains is considered to be an important step in enabling more advanced computational analyses towards understanding tissue properties (biomechanical or architectural, cell subtyping, cellular networks). Furthermore, the use of thicker slices was found to improve the 2D registration by avoiding major distortions, thereby facilitating the combination of information from the slices to construct a meaningful picture for subsequent analyses [2].

**Figure 1.**
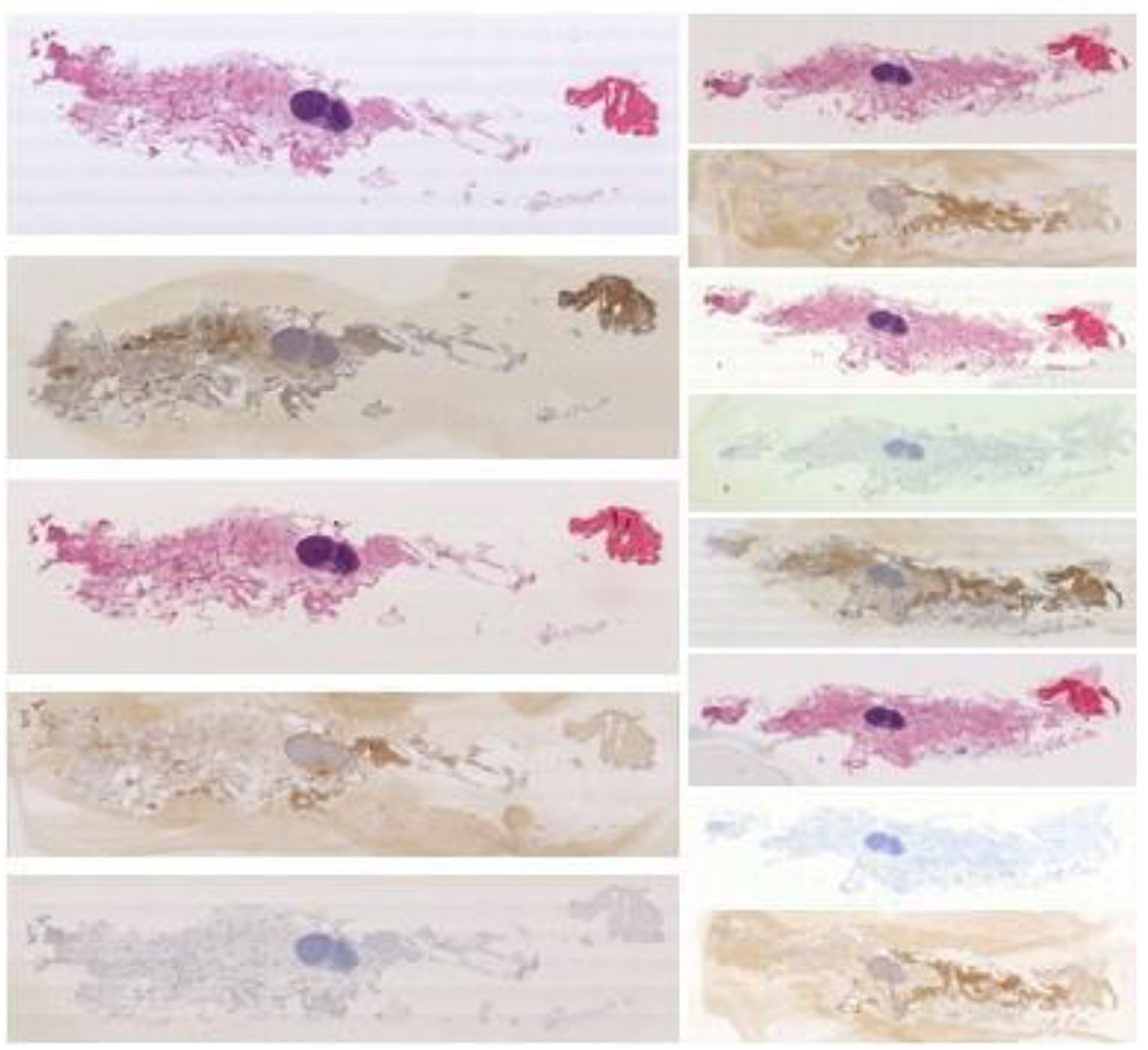
Example mammary gland digitized sequential differently stained histologic whole slide images, as provided by the automatic non-rigid histological image registration (ANHIR) challenge. Figure taken from anhir.grand-challenge.org, last accessed: 13 May 2020.

Various approaches have been proposed for 2D non-rigid registration of histology slides of the same anatomical site, such as B-splines and common information extraction [3], or multiresolution supervised registration [4], on the basis of the *elastix* toolbox [5]. Both these examples [3,4] have reported relatively accurate results in a decent amount of time, but none of them were fully automatic and their evaluation datasets were very small, i.e., 8 pairs of lung histology slides with few different stains [3] and 10 histology slide pairs stained with hematoxylin and eosin (H&E) and anti-PD-L1 antibody (CD274) [4]. Borovec et al. [6] used a comparatively larger multi-stain 2D histologic dataset (Figure 1) to evaluate 11 image registration methods, including intensity-based (*elastix* [5], ANTs [7,8], NiftyReg [9], bUn-warp [10], Multistep [11], DeepHistReg [12]), integral projection-based [13], homography-based [14], feature-based (OpenCV [15], TrakEM2 [16]), hybrid of feature and intensity-based (DROP [17], feature-based + Elastix [18], register virtual stack slices [10]), as well as segmentation-based (ASSAR [19], SegReg [20]) approaches. Some of these approaches were developed during the automatic non rigid histological image registration (ANHIR) challenge and some were developed after the challenge concluded. According to that evaluation study [6], the method with the optimal accuracy and robustness for elastic registration was ANTs [7,8], but at the cost of a very long runtime. An unsupervised registration approach for H&E slides has also been developed on the basis of deep learning features [21–24], reporting relatively good performance with very low runtime. Although such approaches could be applied for computer-assisted interventions [25], they are limited by their need for very large datasets to be efficiently trained and their requirement for specialized hardware (i.e., a general-purpose graphical processing unit (GPGPU)) to achieve low runtime.

## 2. Materials and Methods

### 2.1. Data

To quantitatively evaluate the proposed method, this study used the publicly available data of the ANHIR challenge [6]. ANHIR describes a publicly available multi-institutional dataset [6,26–29] and a community benchmark to fairly evaluate and compare various non-rigid registration methods.

**Figure 2.**
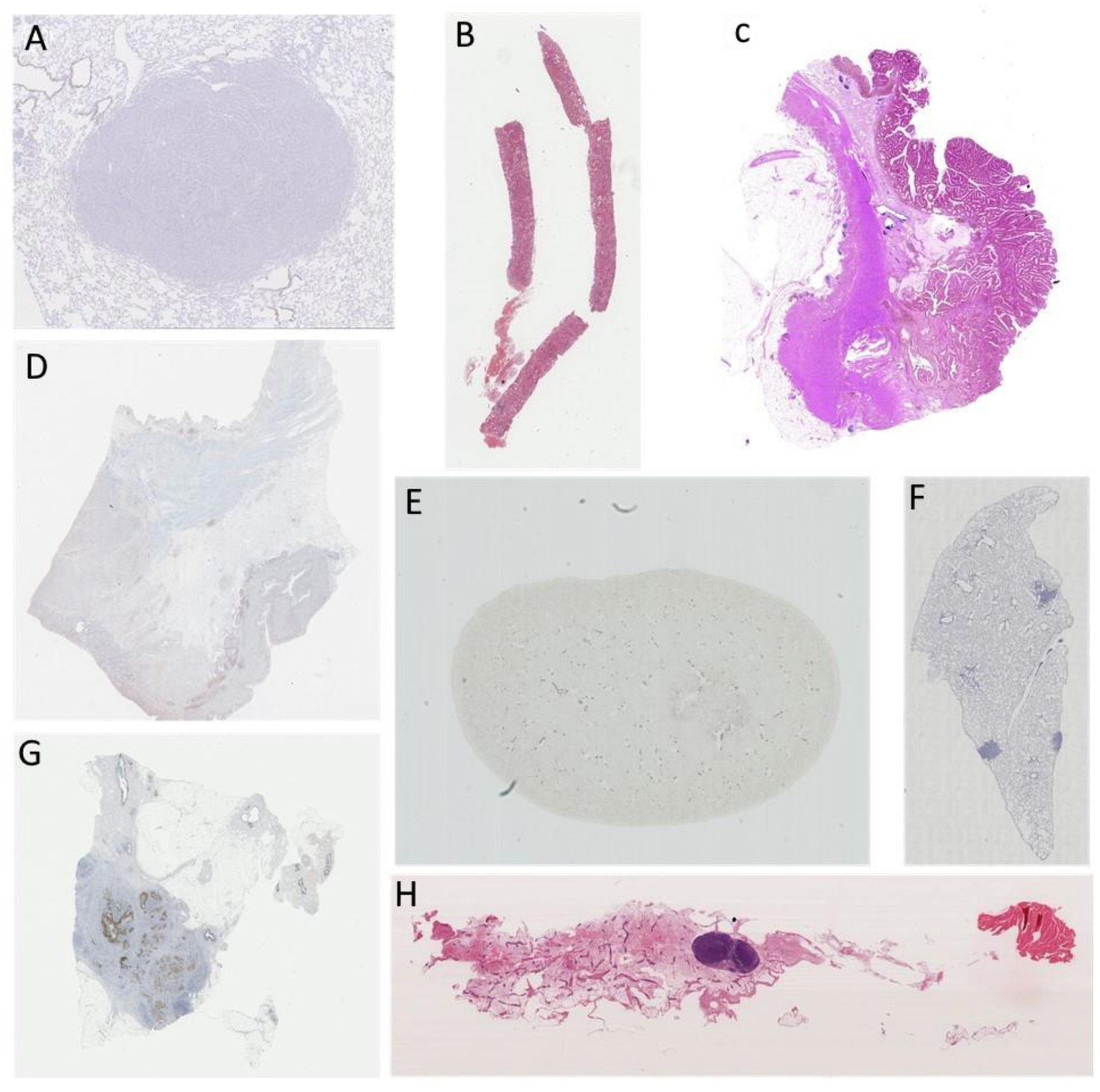
Example histologic images from the various anatomical sites included in the ANHIR dataset, i.e., (**A**) lung lesion, (**B**) kidney, (**C**) colon adenocarcinoma, (**D**) gastric, (**E**) mice kidney, (**F**) lung lobes, (**G**) breast, and (**H**) mammary gland.

ANHIR makes available a set of 481 high-resolution (up to 40× magnification) whole-slide images (n_public_ = 230, n_private_ = 251) from different anatomical sites with manually demarcated landmarks (Figure 2). Specifically, these anatomical sites comprise (*i*) mice lung lesion tissue samples from formalin-fixed paraffin-embedded (FFPE) sections, (*ii*) mice lung lobes corresponding to the same set of histologic samples as the lesion tissue, (*iii*) mammary glands, (*iv*) colon adenocarcinoma, (*v*) resected healthy mice kidneys that show high similarity to human kidneys, (*vi*) surgical material from patients with a histologically verified diagnosis of gastric adenocarcinoma, and (*vii*) FFPE sections of breast and (*viii*) kidney tissue. The original size of the provided images varied from 15 K × 15 K pixels, going up to 50 K × 50 K pixels. However, the images provided for the ANHIR challenge and therefore used to evaluate the performance of our approach represent a scaled version of the original images, of approximately 8 K × 8 K–16 K × 16 K pixels. More than 50 whole-slide histologic image sets were provided and were organized in sets of consecutive sections of the same tissue block of a distinct anatomical site, and each slice was stained with a different dye. The 10 different dyes used in the given dataset were hematoxylin and eosin (H&E), antigen KI-67 (MKI67), platelet endothelial cell adhesion molecule (PECAM1, also known as CD31), estrogen receptor (ESR), progesterone receptor (PGR), human epidermal growth factor receptor 2 (ERBB2), secretoglobin family 1A member 1 (SCGB1A1, CC10), propeptide of surfactant protein C (pro-SFTPC), cytokeratin, and NPHS2 (podocin).

### 2.2. Color Deconvolution

The mammary gland slides stained for ESR and ERBB2 include diaminobenzidine (DAB) stain, which has a brown-dominating appearance and oftentimes significant background staining that makes it very distinct from all other stained slides. Therefore, the hereby proposed approach applies color deconvolution [30,31] only to these slides to distinctly separate the color components of the original images into artificially reproduced *DAB-*, *FastRed-*, and *FastBlue*-stained slides. The intention of this deconvolution is to avoid potential mis-registrations and increase the ability to better assess the underlying tissue structure by lowering the brown-dominating background artefactual appearances from the DAB stain.

**Figure 3.**
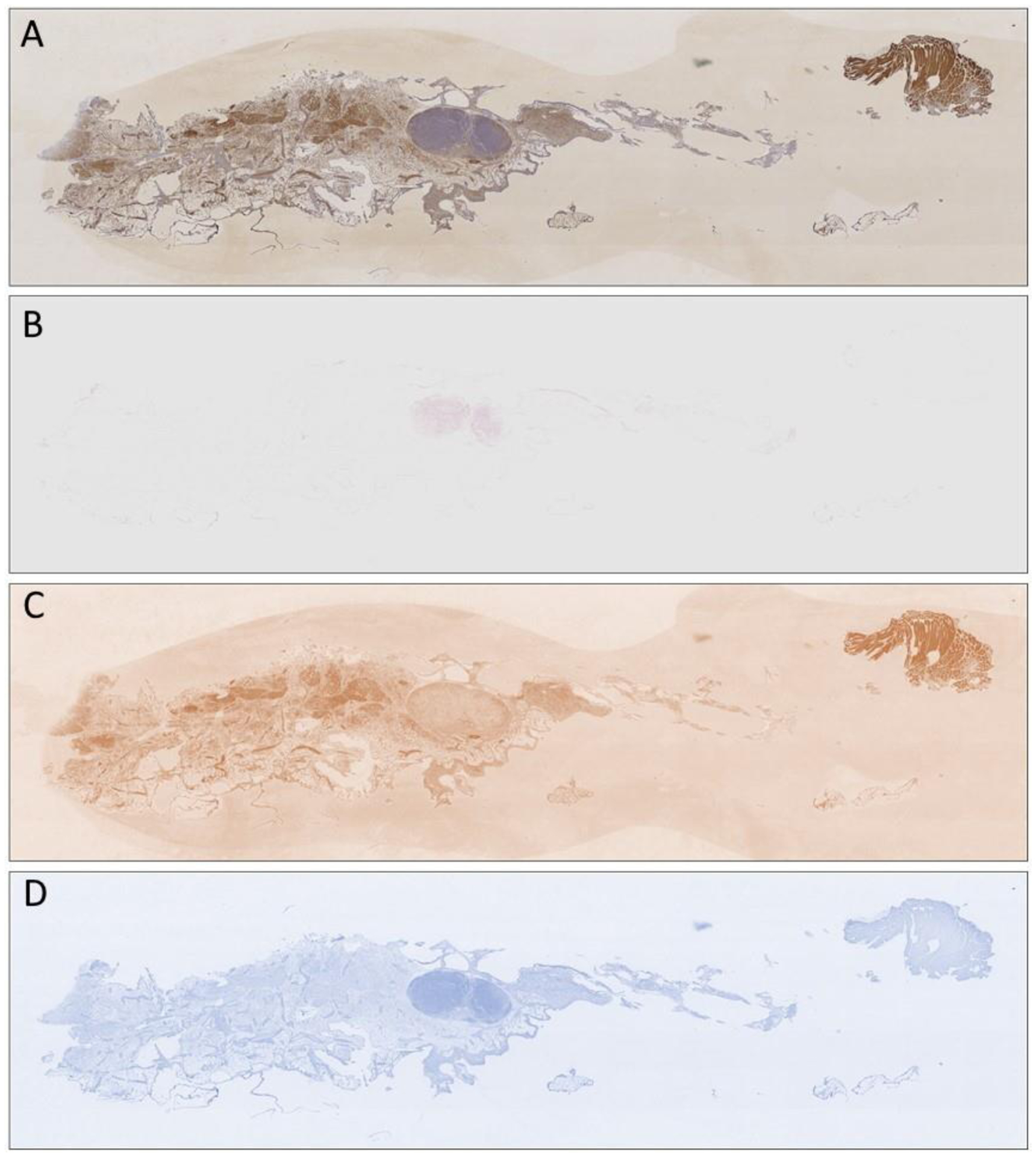
Example results of the applied color deconvolution in a diaminobenzidine (*DAB*)-stained slide, illustrated in (**A**) color deconvolution artificially reproducing and separating the contributions of (**B**) *FastRed*, (**C**) *DAB*, and (**D**) *FastBlue* stains.

An example of this process is shown in Figure 3, where this method was used to artificially reproduce and separate the individual contributions of the *DAB*, *FastRed*, and *FastBlue* stains from the original image. Specifically, the optical density (OD) of the *DAB*, *FastRed*, and *FastBlue* stains are decomposed in their red (R), green (G), and blue (B) channels. Each OD vector is then normalized by its total length, such that each stain forms a normalized RGB triplet. In our case, the OD matrix representing the set of triplets for *FastRed*, *FastBlue*, and *DAB* stains is represented as

(1) $$\begin{array}{cccc} & R & G & B\\ \;\text{Fast}\;Red\; & 0.2140 & 0.8517 & 0.4782\\ \text{Fast}\;\text{Blue} & 0.7489 & 0.6062 & 0.2673\\ DAB & 0.2681 & 0.5703 & 0.7764 \end{array}$$

The color deconvolution matrix is the inverse of this OD matrix and, as detailed in [30], it expresses the mechanism to obtain the corrected contribution of each artificially reproduced stain to the overall image, as if the image was stained using all of them. Here, the color deconvolution matrix is calculated as

(2) $$\begin{array}{cccc} & R & G & B\\ \text{Fast}\;Red & -1.3283 & 1.6219 & 0.2597\\ \text{Fast}\;\text{Blue} & 2.1280 & -0.1584 & -1.2561\\ DAB & -1.1044 & -0.4437 & 2.1210 \end{array}$$

Each row of this matrix represents the factor of the relevant channel/column in the original image that best approximates the contribution of the relevant artificially reproduced stain to the overall image. Negative signs denote information getting subdued and positive signs denote amplification. For example, to obtain the contribution of the *FastBlue* stain, we must subdue portions of the G and B channels by factors of −0.1584 and −1.2561, respectively, while amplifying the R channel by a factor of 2.1280.

As shown in the example results of Figure 3, we observed that the contributions of the artificially reproduced *FastBlue* stain (Figure 3C) retained all the tissue structure information while omitting the background brown-dominating artefact due to the DAB stain (Figure 3D). Therefore, we decided to keep the contribution of the *FastBlue* stain to estimate the transformation between the given consecutive slides.

### 2.3. Pre-Processing

**Figure 4.**
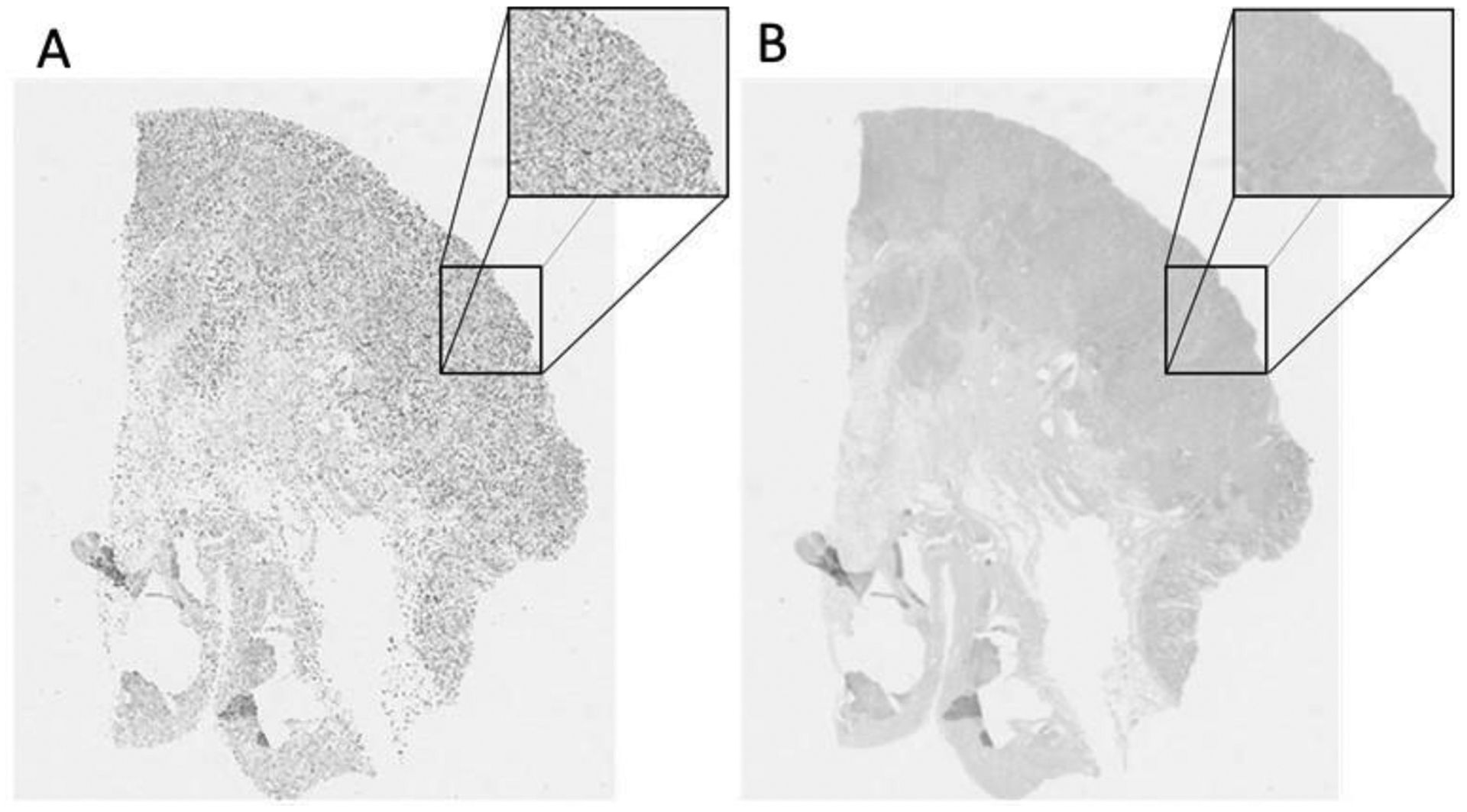
Gastric image resampled before (**A**) and after (**B**) smoothing with a Gaussian kernel.

As the method that was used for this study was originally designed for radiological (specifically, magnetic resonance) images, the histology images needed to be processed to make the characteristics like them. Firstly, taking into consideration the large size of the images used in the evaluation of the proposed approach, we resampled the images on the basis of a factor (*f*) of 1/25 (4%), resulting in image sizes where the minimum and maximum size of each side was between 200 and 700 pixels, respectively, which made the size like that of radiological images, and also helped reduce computation time and memory requirement. To prevent potential aliasing caused due to the large resampling factor, we smoothed the images using a Gaussian kernel (σ = *f*/2) before resampling (Figure 4). The size of the Gaussian kernel was chosen using the Nyquist–Shannon sampling theorem [32], according to which if we want to preserve the invertibility of a transform, the sampling frequency needs to be at least twice the highest frequency of a signal, thereby ensuring that smoothing occurs without loss of structural information within the tissue region.

Furthermore, noting that the provided pairs of images were of varying sizes, we padded each image to ensure that (i) the size of paired images were the same (this step is not mandatory but simplifies the application of the transformation on landmarks) and (ii) the target tissue was in the image center. Once all image pairs were padded such that they were of the same size, we further padded them (4× the size of the similarity metric’s kernel, Equation (1)) to ensure that the apparent tissue was far enough from the image boundaries, and hence accommodated appropriate calculation of the deformation field after changes caused by the affine registration step. A binary mask, computed by excluding the padded portions of the image (size of the similarity metric kernel, Equation (1)) was also used during the affine registration process.

**Figure 5.**
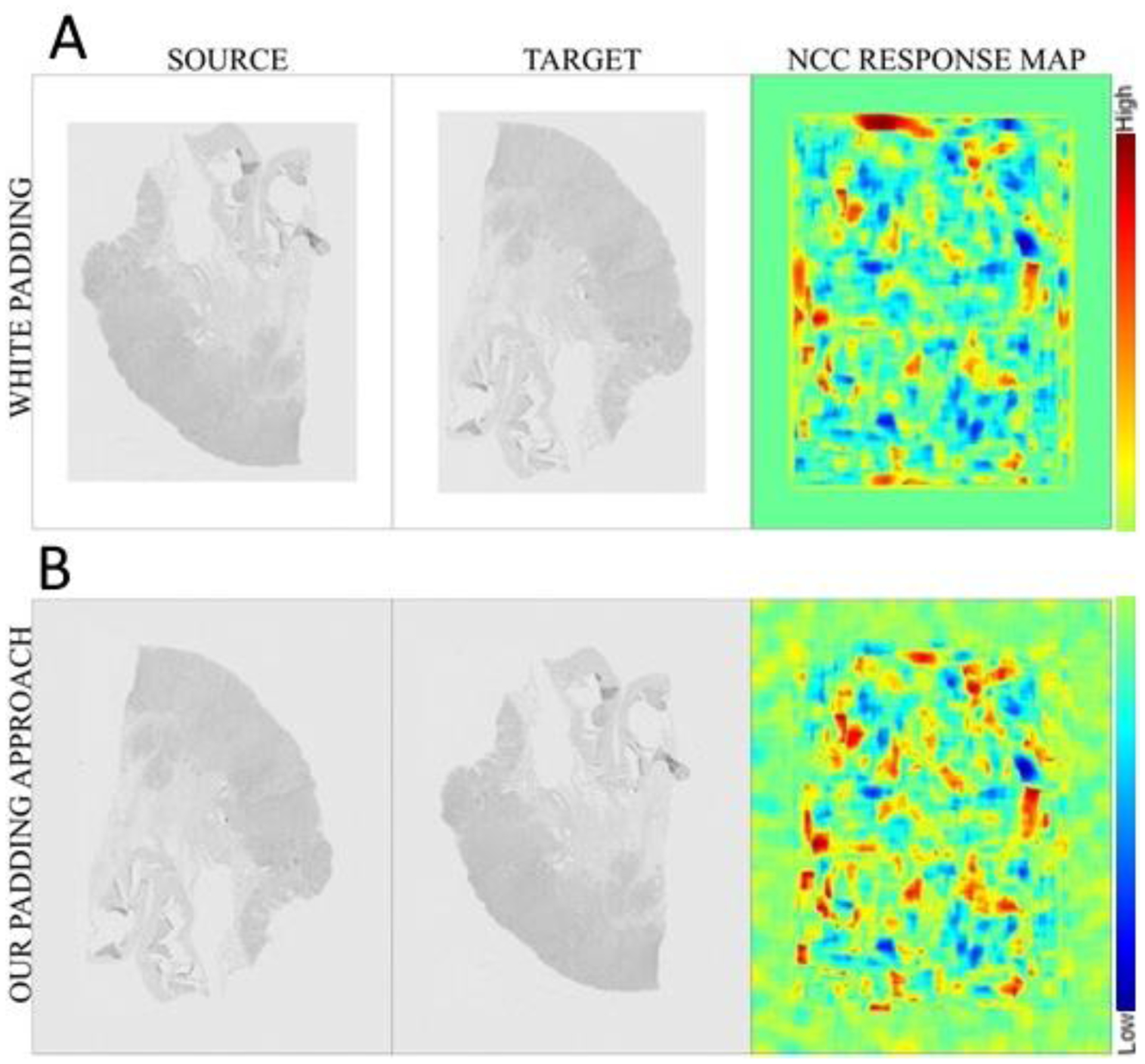
Example results on the difference of the normalized cross-correlation (NCC) response maps after applying white padding (**A**) and our padding approach (**B**). In the top row, the yellow box is noted due to the gradient between the image’s gray background and the white added padding. Conversely, in the bottom row, where the intensities of the four image corners were used for padding, there were no square responses in the NCC. The background NCC responses (due to the added noise) were negligible.

The mask defined the area that computations should be performed, which resulted in improved computational efficiency and no mismatches in terms of boundaries. The padded areas were filled with Gaussian noise matched to the distribution of image intensity in the 4 corners (the size of the similarity metric kernel, Equation (1)) of the unpadded image, which lowered the response of the normalized cross-correlation (NCC) metric along the border between slide background and the padded area (Figure 5).

### 2.4. Registration

For registering the variously stained histologic images, the proposed method adapted “*Greedy*” (github.com/pyushkevich/greedy, hash: 1a871c1, Last accessed: 27 May 2020) [33], a central processing unit (CPU)-based C++ implementation of the greedy diffeomorphic registration algorithm [34]. *Greedy* is integrated into the ITK-SNAP (itksnap.org, version: 3.8.0, last accessed: 27 May 2020) segmentation software [35,36], as well as the Cancer Imaging Phenomics Toolkit (CaPTk—www.cbica.upenn.edu/captk, version: 1.8.1, last accessed: 11 February 2021) [37–39].

*Greedy* shares multiple concepts and implementation strategies within the SyN tool in the ANTs package [7,8] while focusing on computational efficiency by eschewing the symmetric property of SyN and utilizing highly optimized code for computation of image similarity metrics such as NCC, normalized mutual information (NMI), and sum of squared differences (SSD). For the NCC metric, an optimized implementation was used here on the basis of the sum-table algorithm [40]. In general, deformable registration does not do well with the NMI kernel since there are too many degrees of freedom to reduce the dissimilarity metric, i.e., the algorithm can reduce join entropy by non-realistic deformations. Since NCC uses patches, it is much more constrained to match corresponding anatomical locations, thus allowing us to focus on using NCC with an adaptive kernel size scaled with respect to the fixed image size for both the proposed method:

(3) $$\text{ NCC Kernel Radius }=\left\lfloor \frac{\text{Size}\left(I_{i}\right)}{S}\right\rfloor$$

where *S* is the scale by which the width of the fixed image *I*_*i*_ prior to padding is scaled, such that the NCC kernel can pick up enough information for a good registration. After cautious qualitative analysis using various value ranges for *S:* {10, 20, … , 60}, we decided to empirically choose *S* = 40 for both the affine and deformable registration, while optimizing for computational efficiency and accuracy. It is also worth noting that further experimentation with fixed kernels (i.e., 4 × 4 × 4, and 5 × 5 × 5 corresponding to the radius in different scales) resulted in comparable results.

All registrations were performed in a configuration of a multi-resolution pyramid comprising 3 different scales. Specifically, initial registrations were performed on images subsampled by factors of 2^*k*^, and continuous refinements were conducted on images subsampled by factors of 2^*k*−1^, until the final registration occurred at the full resolution images (resolution subsampling factors of 4, 2, 1 were chosen). This process ensures that the most computationally expensive deformations happen at the coarsest resolutions, thereby reducing the overall time and memory requirements. In this paper, the following notation was used:

(4) $$T_{i\to j}^{*}=R\left(I_{i}\to I_{j};\theta \right)$$

where ($T_{i\to j}^{*}$) describes the transformation between fixed (*I*_*i*_) and moving (*I*_*j*_) image, and *θ* defines the registration parameters yielding transformation $T_{i\to j}^{*}$. *R* defines a minimization process such that Equation (2) is unfolded as

(5) $$T_{i\to j}^{*}=\underset{T_{i\to j}}{\text{argmin}}\mu \left(I_{i},I_{j}\circ T_{i\to j}\right)+\lambda \rho \left(T_{i\to j}\right)$$

where *μ* is the similarity metric (SSD, NMI, or NCC, the latter with the kernel size, for instance, NCC[3 × 3]), *λ* is a scalar parameter, and *ρ* is an optional regularization term.

**Figure 6.**
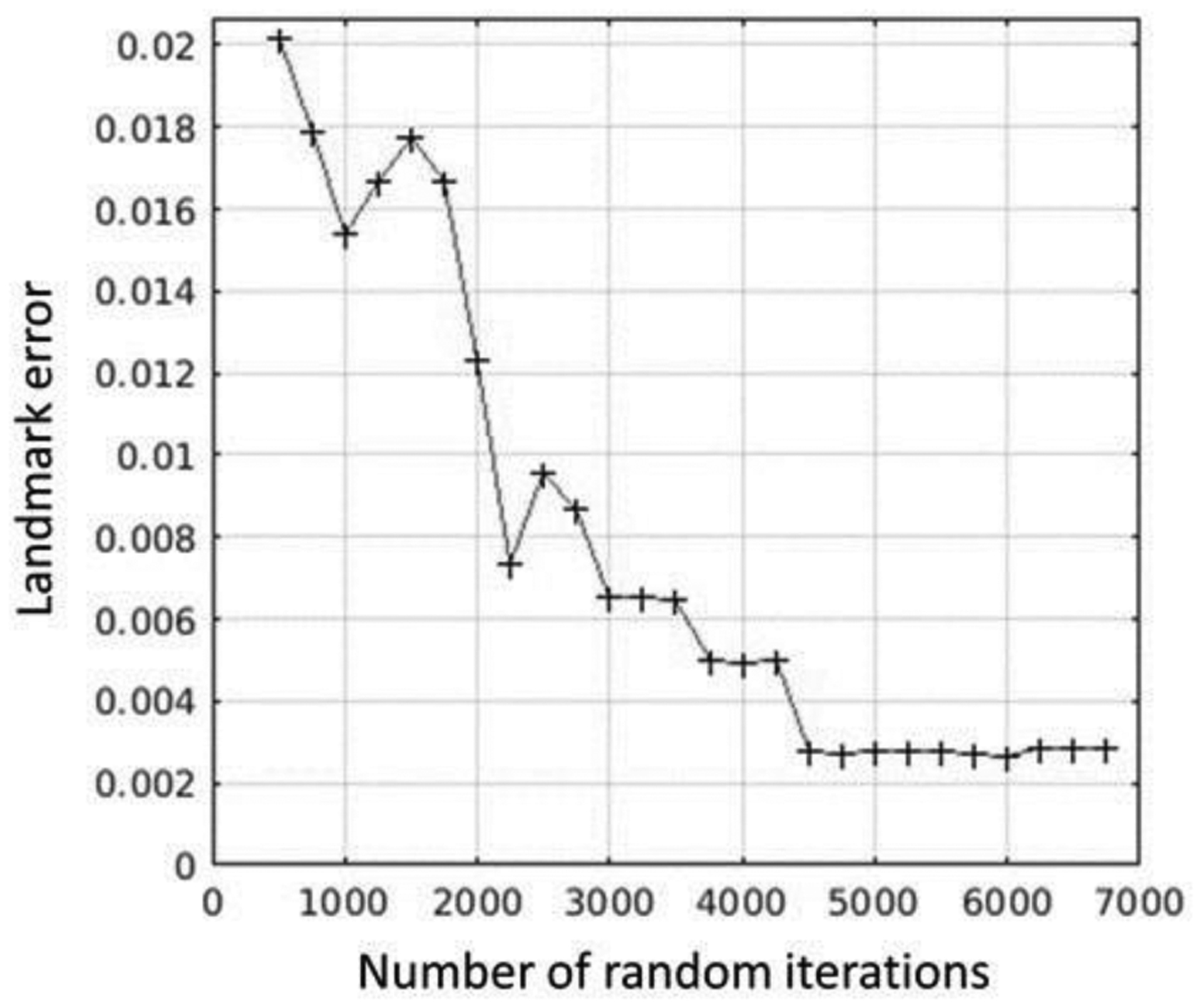
Landmark error over the number of random iterations for the initial transformation.

**Figure 7.**
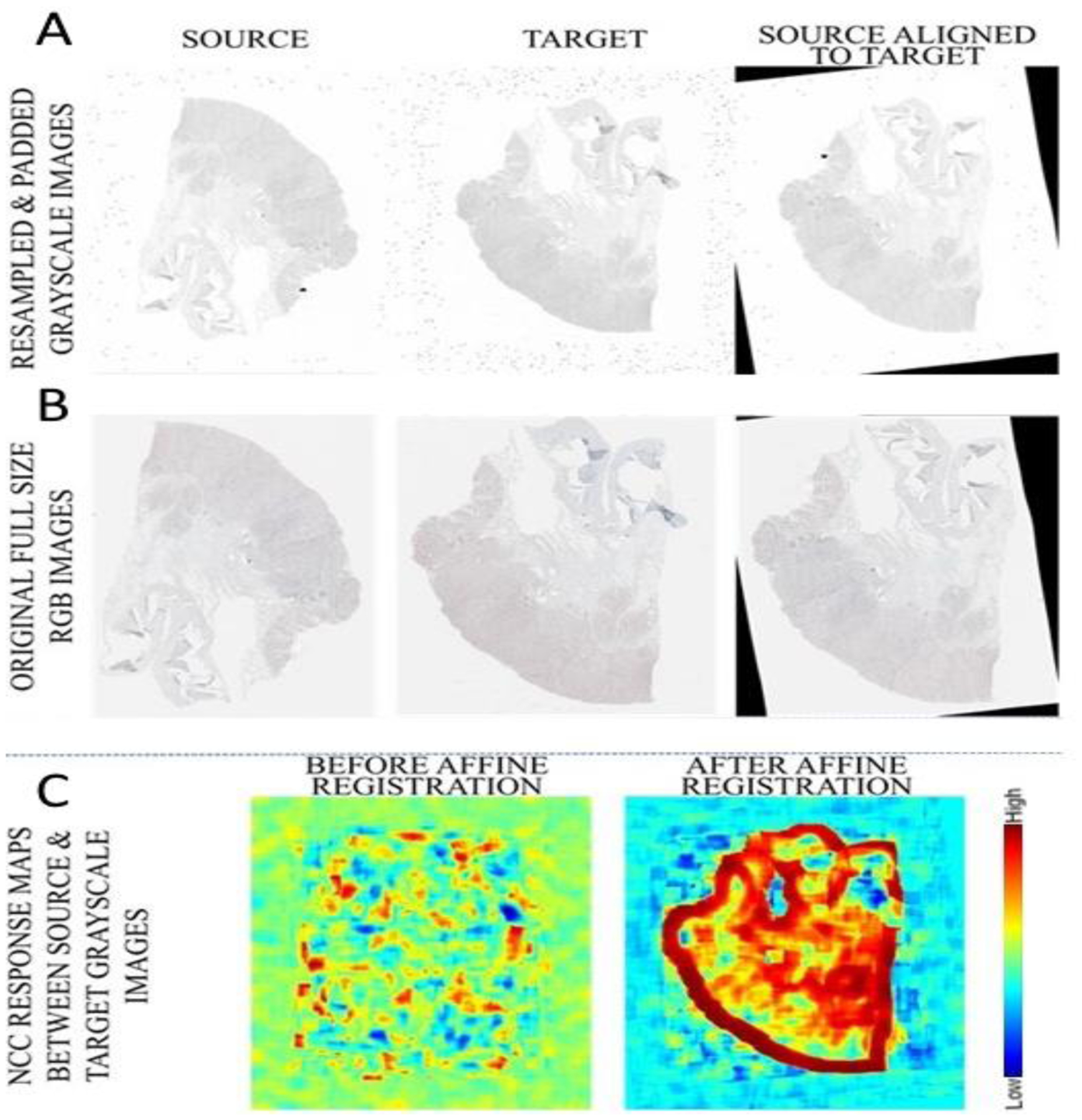
Example results of our affine registration step. (**A**) The affine registration estimated and applied on the resampled and padded images; (**B**) application of the registration applied to the original full-scale images; (**C**) the NCC response map between source and target before and after affine registration.

Initially, affine registration was performed between the image pairs, using an optimization of the dissimilarity metric based on a limited-memory Broyden–Fletcher–Goldfarb–Shanno (L-BFGS) algorithm [41], denoted by

(6) $$A_{i\to j}=R_{aff}\left(I_{i}\to I_{j};\mu ,A_{0}\right)$$

where *A*_0_ is the initial rigid transformation between the images. The initial transformation was obtained using a brute force search, where 4500 pairs of rigid transformations (which captured all possibly combinations of random rotations and translations for the specific dataset) were applied to the moving image and the combination, and the best NCC metric value was saved as *A*_0_. Specifically, a standard deviation of 180° for the angle (ensuring all rotations are sampled) and the standard deviation of the random displacement in each coordinate was equal to 10% of the input image width used, which was large enough to showcase deformation but at the same time small enough to mitigate folding in the dataset due to extreme deformations. Figure 6 illustrates the error in the landmarks as a function of the number of random iterations for the given dataset. This brute force search, performed at the highest pyramid level and not requiring computation of metric gradients, had significant impact on robustness and was relatively fast, i.e., contributing only a few seconds to the total registration time. Figure 7 illustrates an example result from the application of these steps before the actual affine registration.

Following the affine registration, the diffeomorphic registration of slice *j* to *i* was applied:

(7) $$\varphi_{i\to j}=R_{\text{di}\;f\;f\;}\left(I_{i}\to I_{j};\mu ,\sigma_{s},\sigma_{t},N\right)$$

where *σ*_*s*_ and *σ*_*t*_ are the regularization parameters for the registration and *N* is the number of iterations required at each multi-resolution pyramid, e.g., *N* = {100,50,10} refers to 100 iterations at 4×, 50 at 2×, and 10 at full resolution. Larger values of *σ*_*s*_ result in more smoothing, and larger values of *σ*_*t*_ amount to less overall deformation.

Furthermore, *Greedy* uses an optimized smoothing of the deformation fields on the basis of the ITK recursive Gaussian smoothing classes [42]. The actual registration was computed in an iterative manner using the update equations [43]:

(8) $$\psi^{\gamma }=Id+\varepsilon^{\gamma }\cdot \left[G_{\sigma_{S}}*D_{\varphi_{i\to j}^{T}}\mu \left(I_{i},I_{j}\;o\;\varphi_{i\to j}^{\gamma }\right)\right]$$

(9) $$\varphi_{i\to j}^{\gamma +1}=G_{\sigma_{t}}*\left(\varphi_{i\to j}^{\gamma }\;o\;\psi^{\gamma }\right)$$

(10) $$\varphi_{i\to j}^{0}=Id$$

where *γ* is the current iteration, $D_{\varphi_{i\to j}^{T}}\mu$ is the gradient of the metric with respect to *φ*, *ε*^*γ*^ is the step size, *G*_*σ*_ * *φ* denotes the convolution of *φ* with an isotropic Gaussian kernel with a standard deviation of *σ*, and *Id* is the identity transformation. For sufficiently smaller *ε*^*γ*^ and larger *σ*_*s*_ values, *ψ*^*γ*^ is smooth and has a positive Jacobian determinant for all *x* ∈ Ω_*i*_, thereby making the registration diffeomorphic in nature. As diffeomorphisms form a group under composition, $\varphi_{i\to j}^{T+1}$ is also diffeomorphic in nature [34].

These registration steps result in 2 matrices describing the affine and deformable transformations, from target to source images. To apply these transformations in the ANHIR data, we first mapped the original manually demarcated landmarks into the down-sampled and padded image space, then we applied the computed inverse transformation, and finally we mapped the transformed landmarks back to the original resolution space.

### 2.5. Evaluation

The performance of our method was quantitatively evaluated on the basis of landmarks provided by the challenge organizers. Specifically, the quantitative performance evaluation framework reported here is consistent with the one used during the ANHIR 2019 challenge (anhir.grand-challenge.org/Performance_Metrics/, last accessed: 13 May 2020) and it was based on the metrics of (a) the average of the median relative target registration error (*rTRE*) and (b) the robustness (*R*) criterion. Notably, the benchmarking framework to calculate these metrics is available in borda.github.io/BIRL, as provided by the ANHIR challenge organizers [6]. Since the challenge participants did not have access to neighboring slices, the challenge organizers had asked for pairwise registration and not the complete 3D reconstruction of the tissue to generate the aforementioned metrics.

*rTRE* represents the geometric accuracy between the target and warped landmarks in the target image frame. The motivation for using the median is to avoid penalization of few inaccurate landmarks, especially when the others are well-registered. Since only the challenge organizers had access to the testing dataset of the challenge for obvious reasons, in this study, results based on the *rTRE* achieved in the public data of the ANHIR challenge are reported. Specifically, *TRE* is defined as

(11) $$TRE=d_{e}\left(x_{l}^{T},x_{l}^{W}\right)$$

where $x_{l}^{T}$ and $x_{l}^{W}$ are the coordinates of the landmarks “*l*” in the target and warped image, and *d*_*e*_(.) defines the Euclidean distance. All *TRE* are then normalized by the diagonal of the image to define the *rTRE*:

(12) $$\text{rTRE}=\frac{TRE}{\sqrt{w^{2}+h^{2}}}$$

where *w* and *h* denote the image’s width and height, respectively.

The proposed approach was also evaluated according to the metric of robustness (*R*), which takes values in the range of 0 and 1. When *R* is equal to 1, the average distance of all the landmarks in the moving and fixed images is reduced after registration (defining the absolute algorithmic robustness), and 0 means that none of the distances are reduced. The mathematical formulation of *R* for the *i*^th^ image of the dataset marked with *L*_*i*_ landmarks is a defined as

(13) $$R_{i}=\frac{1}{L_{i}}\sum_{j\in L_{i}}\left(rTRE_{j}^{\text{regist}}<rTRE_{j}^{\text{init}}\right)$$

where $rTRE_{j}^{\text{init}}$ is the *rTRE* of the *j*th landmarks initially and $rTRE_{j}^{\text{regist}}$ is the *rTRE* after registration. *R* is therefore a relative value of how many landmarks have an improved *rTRE* after registration.

It is worth noting that the ranking of the ANHIR challenge was not based on the absolute *rTRE* and *R* metrics, but on the relative performance considering all participating teams. This was obtained by averaging the ranked *rTRE* scores (unavailable for participants) across each pair of images.

## 3. Results

**Figure 8.**
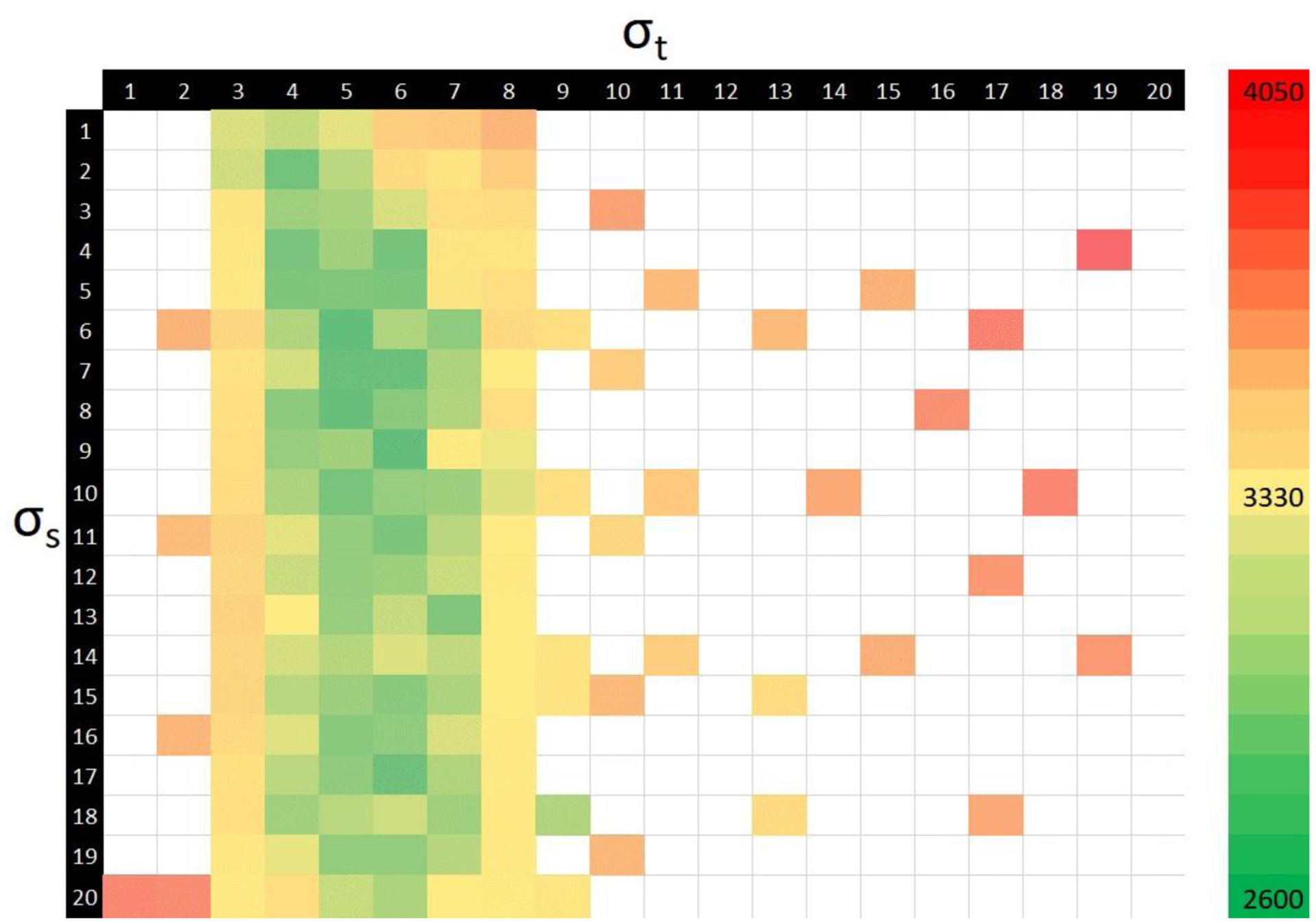
Heatmap showcasing the average error rate for different combinations of for *σ*_*s*_ and *σ*_*t*_ (lower is better).

The proposed approach used the public data alone to perform a grid search (i.e., perform an exhaustive search across the various parameter combinations using pre-defined steps to ascertain the optimum combination to lower the average error rate) for *σ*_*s*_ and *σ*_*t*_ in the range of [20,20] and found the optimal values to be 6 and 5 pixels, respectively (Figure 8). No parameter tuning was performed on the hold-out dataset.

**Figure 9.**
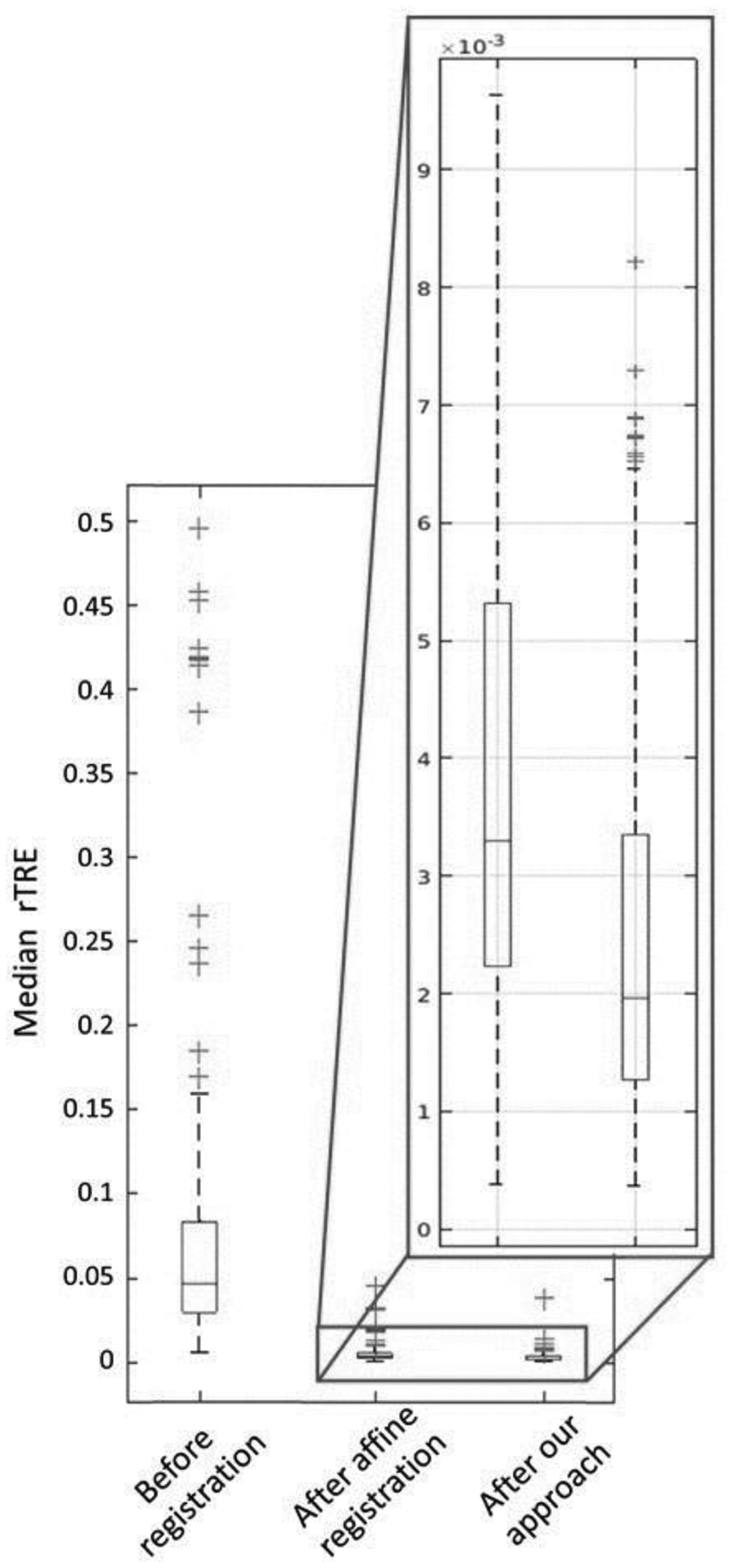
The overall median relative target registration error (*rTRE*) across all public data before any registration, after the affine step, and after both affine and diffeomorphic registration.

**Figure 10.**
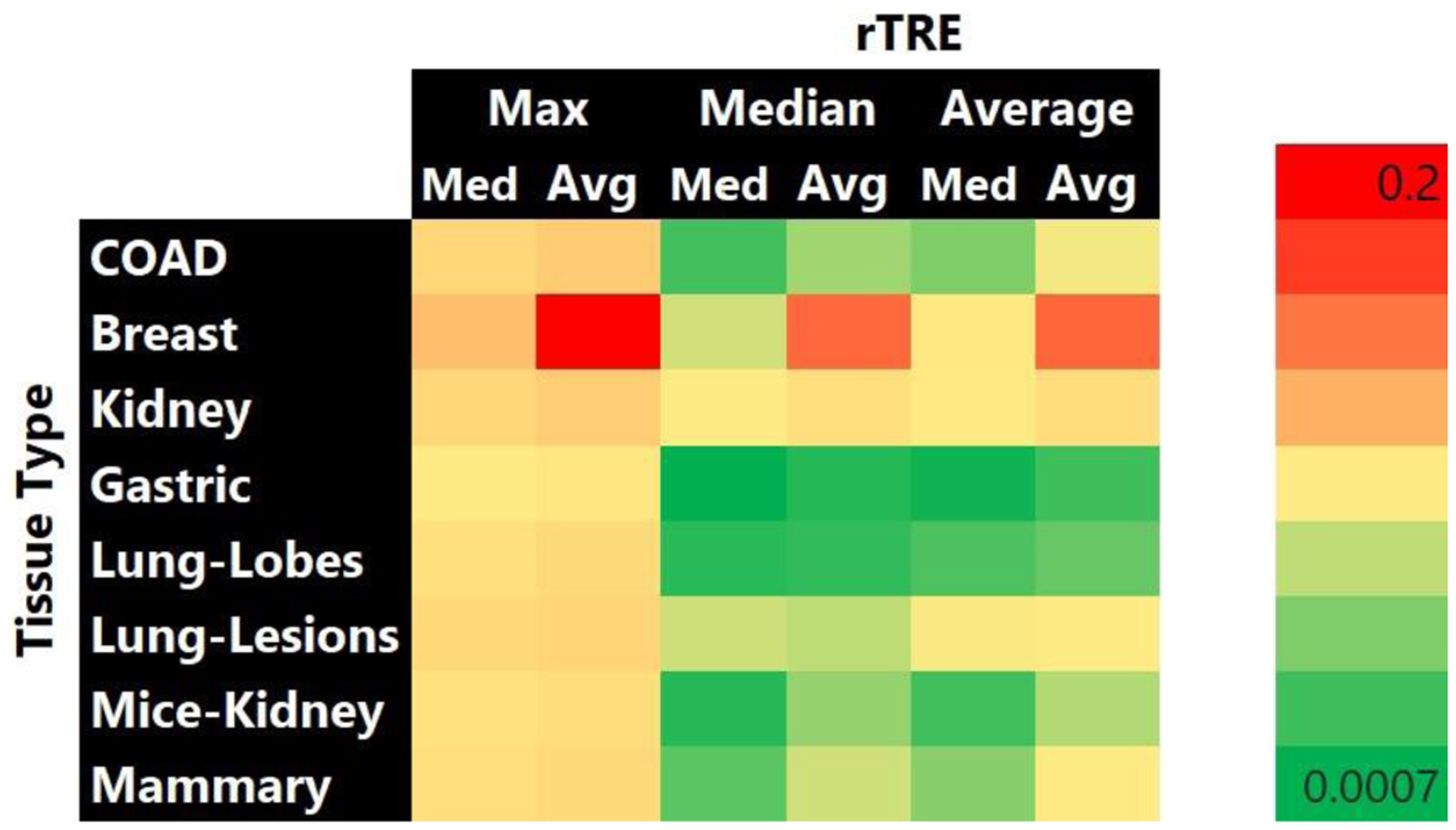
*rTRE* values for various tissue types for the evaluation data using the approach proposed.

**Figure 11.**
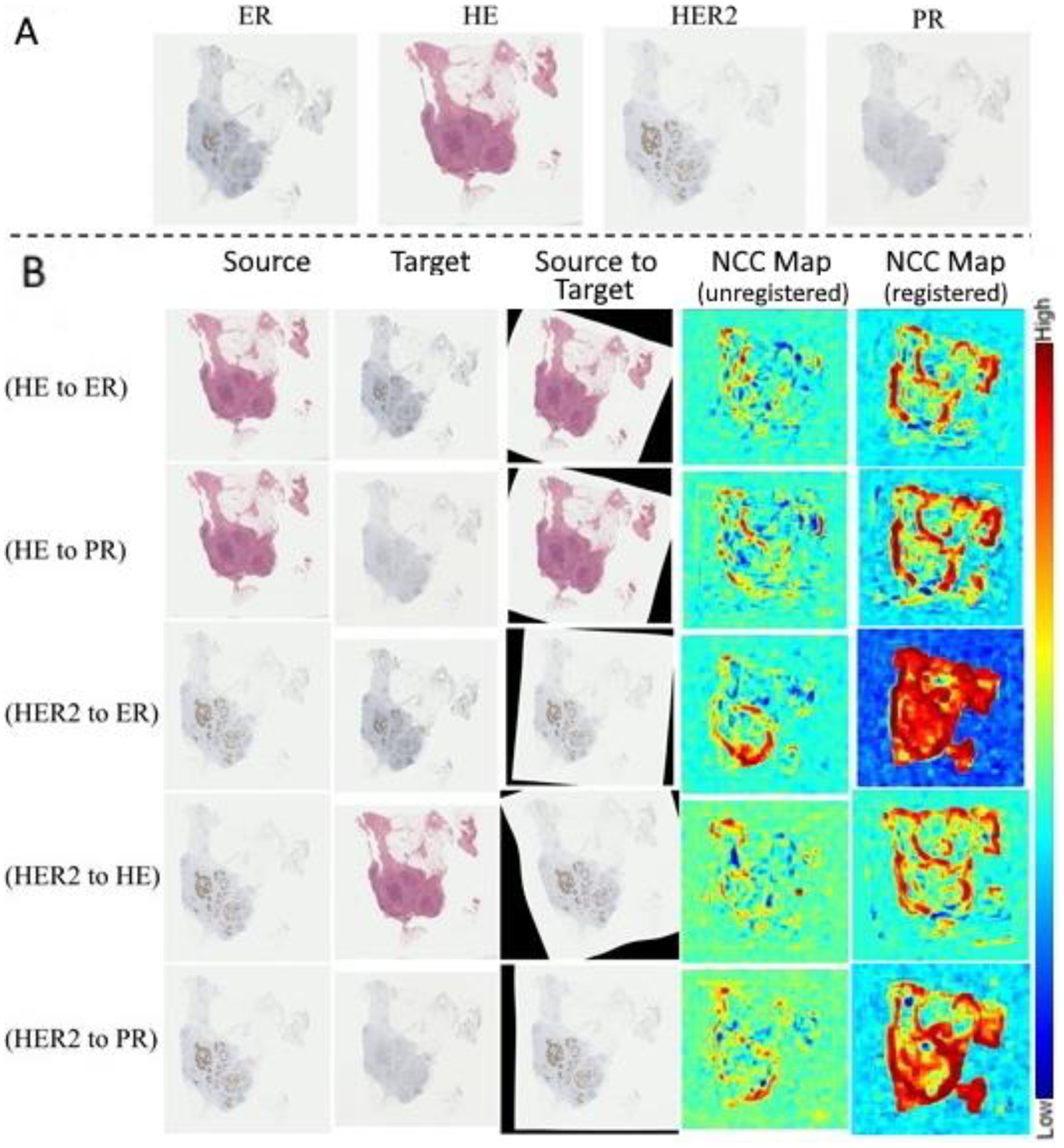
(**A**) Four example consecutive differently stained images from a breast tissue case. Different stains include hematoxylin and eosin (H&E), estrogen receptor (ESR), progesterone receptor (PGR), and human epidermal growth factor receptor 2 (ERBB2). (**B**) Example registration results of the source image, registered to the target, resulting in the aligned source. The NCC response maps before and after registration are illustrated in the two right columns.

**Figure 12.**
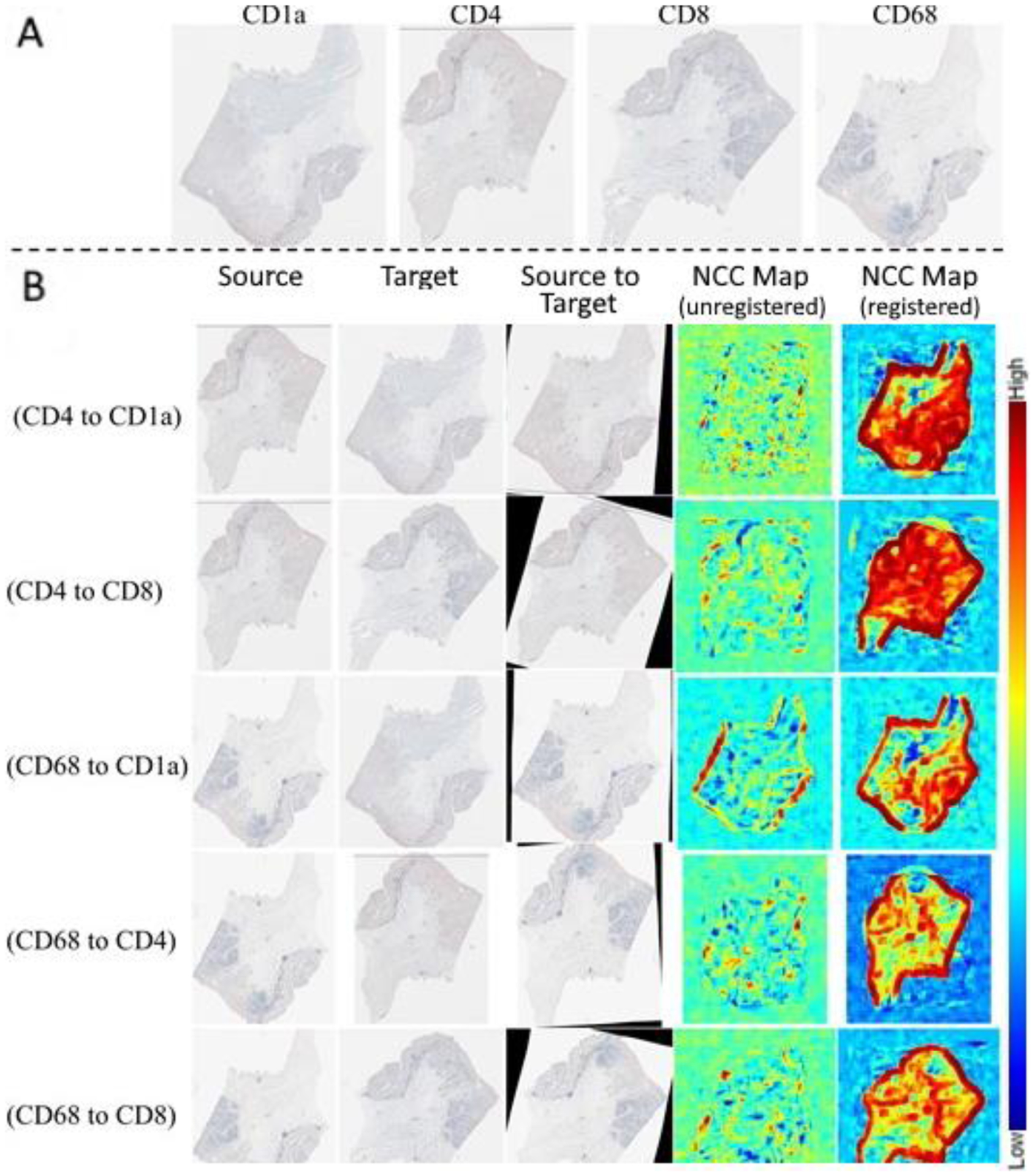
(**A**) Four example consecutive differently stained images from a Gastric mucosa and gastric adenocarcinoma tissue, showing the different stains, namely, CD1A, CD4, CD8, and CD68. (**B**) Example registration results of the source image, registered to the target, resulting in the aligned source. The NCC response maps before and after registration are illustrated in the two right columns.

The averages across all image pairs of the median *rTRE* for the affine and the deformable registration were equal to 0.00473 and 0.00279, respectively (Figure 9). Figure 9 indicates the improvements in the *rTRE* before applying any registration, after applying only affine registration, and after the proposed approach. Notably, when compared to the other participating methods, the proposed method’s (*HistoReg*) score of 0.00279 was the highest score achieved using the public data during the 2019 ANHIR challenge [38] (as indicated in the official challenge webpage: anhir.grand-challenge.org/Workshop-ISBI19/, last accessed: 13 May 2020). It is further noted that the median robustness of the proposed method, as defined by the challenge, was equal to 1 and the average robustness was 0.9898. As shown in Table III of the ANHIR article [38], *HistoReg*’s overall rank during the challenge was 2, on the basis of the median of median *rTRE* values (our score was 0.0019). However, it was the best ranked method when the average or the median of average *rTRE* (score of 0.0029) values and average robustness (0.9898) were the evaluation criteria. Observed discrepancies between the ANHIR publication [38] and the ANHIR’s webpage were attributed to the fact that the challenge organizers allowed submissions to their testing system after the challenge was completed. The overall low *rTRE* values contributed towards proving the overall efficacy of the method, with the notable lowest values coming for Gastric tissue slices and the highest values coming from the breast tissue slides, with the median–median *rTRE* value going as low as 0.0007, and as high as 0.2, respectively (Figure 10). These results represent the best and worst results in the challenge, respectively. Registrations of consecutive differently stained images from two distinct anatomical sites are illustrated in Figures 11 and 12.

It is also noted that depending on the metric used for the final challenge, ranking the methodological performance of the proposed approaches changed. Importantly, the approach presented in this manuscript remains stable for any ranking criterion [44] defined by the challenge organizers, as evidenced by the statistics presented in [44,45].

Finally, the average time needed to compute the registration for a pair of images was equal to 29 s on an Intel Xeon Gold 6130 using eight threads and 32 Gb of RAM. The computation time, which was normalized using the computation time of the evaluation scripts given by the challenge organizers, was equal to 1.45 min.

## 4. Discussion

The hereby study highlights an approach for performing non-rigid registration of variable-stained histologic whole slide images, agnostic to the anatomical site that the slide is sectioned from. Quantitative evaluation on publicly available data of 10 different dyes applied on tissue types from eight distinct anatomical sites, during a community benchmark, sets our proposed methodology in the top two best performing ones. Notably, the proposed approach is as effective on datasets consisting of sequential tissue sections, as it is on non-sequential tissue sections, an important feature given that clinical cases often consist of non-immediate-sequential sections. This can be considered as the first step in allowing downstream assessment of a 3D volume of digitized slides of clinical tissue specimens.

Current routine clinical histopathologic evaluation of disease is based on the microscopic assessment of 2D tissue sample representations. Although 3D tissue evaluation is accepted to offer more contextual information of the disease microenvironment (such as vessel tortuosity), enabling equipment remains part of research laboratories due to associated costs and specialized training. An acceptable schema for evaluating 3D anatomical structure in each dataset is to assess consecutive tissue sections across the *z*-axis (depth of tissue within a paraffin block of tissue). This process empowers the evaluation of the anatomic pathology and histology, as well as of characteristics of multiple markers (protein, RNA, and DNA targets) for a patient in a single tissue area across various sequential sections. It can further enable a pathologist to extract detailed contextual information about the entire section, and, in particular, enable a better understanding of the spatial arrangement and growth patterns of cells and matrix (vessels, stroma, and immune cells) as it relates to tissues and organs. An automated methodology allowing tissue assessment in 3D, while being able to deal with extreme appearance changes and significant background staining (e.g., DAB stain), without requiring any specialized training, but by virtue of associating consecutive routine clinically acquired whole-slide images, is appealing for richer clinical evaluation of anatomic pathology and histology, as well as of characteristics of multiple markers. Furthermore, such a methodology can contribute to the concepts of accountability, explainability, and transparency in computational systems [46,47], as it can assist a clinical pathologist to better understand the spatial arrangement and growth patterns of cells and matrix (vessels, stroma, and immune cells) but also offer a deeper insight for downstream research analysis of specific disease.

This study showed that a general-purpose tool originally developed for registration of 3D radiographic images, such as magnetic resonance imaging (MRI), can achieve excellent performance in the domain of histology registration. *Greedy* has previously been used for histology–MRI matching [43], and no major algorithmic developments were needed to adopt it to this task and challenge. The proposed approach does not require any specialized hardware (i.e., GPU) as it is CPU-based and achieves relatively low computation time by using highly optimized code for similarity metric computations. The code related to the package (including pre-processing) is available through our GitHub page at github.com/CBICA/HistoReg (accessed: 11 February 2021).

Future work related to this study includes more exhaustive performance evaluation of the *Greedy* algorithm and its comparison with alternative approaches, e.g., those based on detection of salient points [48]. Although the scope of this study focused on the registration of consecutive whole-slide images, the overarching goal of this work was to contribute towards reconstruction the 3D anatomical tissue structure from 2D histology slices [43,49,50], irrespective of the staining applied to them, in order to give more context and evaluate the association of anatomical structures in the microscopic scale with the molecular characterization of the associate tissue samples. Notably, this is of interest in cancer, where such associations are already evaluated in the macroscopic scale on the basis of radiographic representations [51–54]. Moreover, the proposed approach could complement databases, such as the one described by Yagi et al. [32], that consider differently stained whole-slide images, and integrating clinical, histologic, immunohistochemical, and genetic information to contribute towards multi-parametric research and aid in pathologic diagnosis by optimizing the effective viewing and evaluation of differently stained whole slide images.

This study has shown that registration of variably stained histology whole-slide images can be performed robustly across tissue types, agnostic to the anatomical site. Furthermore, maintaining computational efficiency without the need of any specialized hardware and ensuring cross-platform compatibility should relate to potentially easier clinical translation. To facilitate this, the implementation of this study has been released as an open-source paradigm, enabling its application in more diverse histological datasets.
